# Severe mesangiolysis with prominent microaneurysm formation following total body irradiation conditioning for bone marrow transplantation

**DOI:** 10.1007/s13730-026-01130-5

**Published:** 2026-05-20

**Authors:** Yosuke Nakagawa, Masahiro Koizumi, Norisuke Shimamura, Naoto Hamano, Go Ogura, Takehiko Wada, Makoto Onizuka, Hirotaka Komaba

**Affiliations:** 1 Division of Nephrology, Endocrinology and Metabolism, Tokai University School of Medicine, 143 Shimokasuya, Isehara, 259-1193 Japan; 2 Department of Nephrology, Shin-Yurigaoka General Hospital, Kawasaki, Japan; 3 Department of Pathology, Tokai University School of Medicine, Isehara, Japan; 4 Department of Nephrology, Toranomon Hospital, Tokyo, Japan; 5 Department of Hematology/Oncology, Tokai University School of Medicine, Isehara, Japan

**Keywords:** Hematopoietic stem cell transplantation, Thrombotic microangiopathy, Radiation nephropathy, Mesangiolysis, Microaneurysm

## Abstract

Patients who undergo hematopoietic stem cell transplantation (HSCT) face a high risk of acute kidney injury (AKI), which significantly increases the likelihood of developing chronic kidney disease (CKD). Proteinuria is also frequently observed after HSCT and is associated with an increased risk of CKD progression. Thrombotic microangiopathy (TMA), stemming from endothelial injury caused by factors such as total body irradiation (TBI), calcineurin inhibitors, tyrosine kinase inhibitors, and graft-versus-host disease, is a major contributor to both AKI and CKD. We report a case of a woman in her early thirties who developed progressive kidney dysfunction and subnephrotic-range proteinuria three months after HSCT. Kidney biopsy findings included marked mesangiolysis, endothelial cell detachment, subendothelial expansion, and diffuse segmental capillary microaneurysms, which are highly suggestive of TMA due to TBI administered as part of the myeloablative conditioning regimen prior to HSCT. She underwent conservative management for chronic kidney disease, but her kidney function progressively declined. In patients presenting with a progressive decline in kidney function following HSCT, TMA should be included in the differential diagnosis. A kidney biopsy is valuable for determining the underlying cause of HSCT-associated TMA, as treatment strategies vary depending on the etiology. Prominent mesangiolysis with capillary microaneurysms, as observed in this case and the previously reported irradiated patients, may serve as a distinctive histological marker of radiation nephropathy.

## Introduction

Patients who undergo hematopoietic stem cell transplantation (HSCT) face a high risk of acute kidney injury (AKI) [1], which significantly increases the likelihood of developing chronic kidney disease (CKD) [2–4]. Proteinuria is also frequently observed after HSCT and is associated with an increased risk of CKD progression [5]. Among the various etiologies of kidney injury in this setting, thrombotic microangiopathy (TMA), characterized by microangiopathic hemolytic anemia, consumptive thrombocytopenia, and microvascular thrombosis secondary to progressive endothelial damage, is a major contributor to both AKI and CKD. A meta-analysis of observational studies published between 2004 and 2020 estimated that the pooled incidence of HSCT-associated TMA (HSCT-TMA) was between 9 and 16% [5]. In patients with slowly progressive kidney impairment with mild to moderate proteinuria following HSCT, TMA should be considered in the differential diagnosis. The causes of HSCT-TMA are multifactorial and may include graft-versus-host disease (GVHD), use of calcineurin inhibitors (CNIs), use of tyrosine kinase inhibitors (TKIs), and myeloablative conditioning regimens involving total body irradiation (TBI) and alkylating agents [6–9]. Since therapeutic strategies differ on the basis of the underlying etiology, kidney biopsy plays an important role in establishing an accurate diagnosis. Here, we report a case of severe mesangiolysis with prominent formation of capillary microaneurysms, likely attributable to TBI administered as a part of the myeloablative conditioning regimen prior to HSCT.

## Case report

**Fig. 1:**
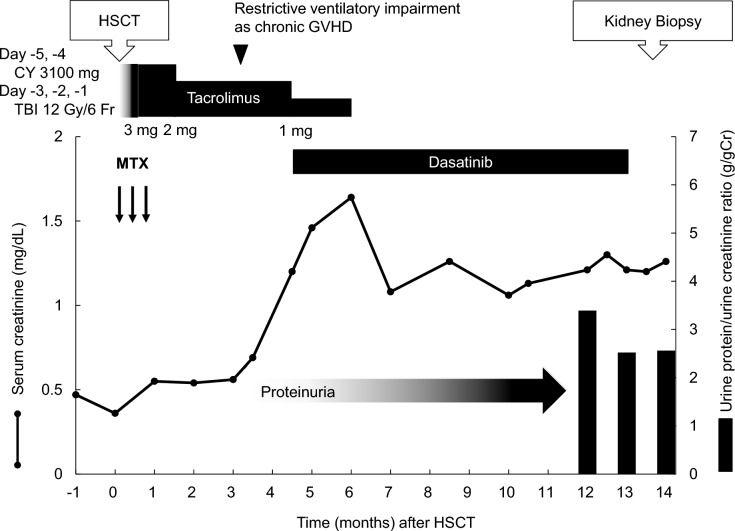
Clinical course after hematopoietic stem cell transplantation. Abbreviations: CY, cyclophosphamide; GVHD, graft-versus-host disease; HSCT, hematopoietic stem cell transplantation; MTX, methotrexate; TBI, total body irradiation

A Japanese woman in her early thirties had been diagnosed with Philadelphia chromosome-positive acute lymphoblastic leukemia two years prior to this presentation. Complete remission was achieved by treatment with cytarabine and daunorubicin followed by dasatinib, a TKI. She subsequently underwent allogeneic HSCT from a matched unrelated donor. The clinical course is summarized in Fig. 1. As part of the myeloablative conditioning regimen, she received intravenous cyclophosphamide on days -5 and -4 and TBI from days − 3 to − 1 (total dose: 12 Gy). Kidney shielding was not performed at the time of irradiation because she had a poor-prognosis malignancy. For acute GVHD prophylaxis, she was administered intravenous methotrexate and tacrolimus, a CNI. She developed no signs of acute GVHD and was discharged on Day 35 posttransplant. One month after HSCT, her serum creatinine level was 0.9 mg/dL, and her urinalysis results were normal. Over the course of three months, she developed persistent nonproductive cough and mild restrictive ventilatory impairment; inhaled corticosteroids were initiated due to suspected chronic GVHD. Around this time, her serum creatinine level increased to 1.2 mg/dL, and proteinuria developed. Despite discontinuation of tacrolimus use, her serum creatinine levels did not improve, and her proteinuria progressively worsened to 2.0–3.0 g/gCr. She was referred to our nephrology department for further evaluation. Her medical history included Graves’ disease in long-term remission after methimazole treatment and radioiodine ablation and secondary amenorrhea treated with Kauffman therapy. Current medications include inhaled salmeterol/fluticasone, estradiol, and dydrogesterone. Her height was 148.9 cm, her weight was 52.4 kg, her blood pressure was 125/93 mmHg, and her pulse rate was 121 beats per minute. She had no prior diagnosis of hypertension. The physical examination was unremarkable. At the time of nephrology consultation, her serum creatinine level was 1.21 mg/dL, her urinary protein excretion was 3.39 g/gCr, and her serum albumin level was within normal limits. Urine sediment revealed granular casts, fatty casts, and oval fatty bodies. ADAMTS13 activity was not measured because the patient lacked laboratory findings suggestive of systemic TMA. The laboratory data are summarized in Table 1.

**Table 1:** Laboratory findings at kidney biopsy

Urinalysis			Complete blood count					
Protein	(2 +)		WBC	7000	/µL	BUN	24	mg/dL
Occult blood	( +/−)		RBC	322 × 10^4^	µL	Cr	1.21	mg/dL
Glucose	(−)		Hb	10.8	g/dL	Na	143	mEq/L
Sediments			Hct	32.4	%	K	4.2	mEq/L
RBC	1–4	/HPF	Plt	20.5 × 10^4^	µL	Cl	107	mEq/L
WBC	1–4	/HPF				Ca	10.0	mg/dL
Granular cast	(1 +)		Blood chemistry			IP	3.4	mg/dL
Fatty cast	(1 +)		AST	23	U/L	Fe	41	µg/dL
Oval fatty body	(1 +)		ALT	28	U/L	TIBC	216	µg/dL
			LDH	205	U/L	Ferritin	917	ng/mL
Protein	3.39	g/gCr	ALP	247	U/L	CRP	0.65	mg/dL
ß2MG	1.29	mg/L	γ-GTP	33	U/L	IgG	613	mg/dL
NAG	7.8	U/L	TP	7.4	g/dL	IgA	87	mg/dL
			Alb	4.6	g/dL	IgM	60	mg/dL
Selectivity index	0.51		T.chol	242	mg/dL	C3	129	mg/dL
			UA	6.4	mg/dL	C4	40.9	mg/dL

Alb, albumin; ALP, alkaline phosphatase; ALT, alanine aminotransferase; AST, aspartate aminotransferase; ß2MG, beta-2 microglobulin; BUN, blood urea nitrogen; Ca, calcium; C3, complement component 3; C4, complement component 4; Cl, chloride; Cr, creatinine; CRP, C-reactive protein; Fe, iron; γ-GTP, gamma-glutamyl transpeptidase; g/gCr, grams per gram creatinine; Hb, hemoglobin; Hct, hematocrit; HPF, high-power field; IgA, immunoglobulin A; Ig, immunoglobulin; IP, inorganic phosphate; K, potassium; LDH, lactate dehydrogenase; Na, sodium; NAG, N-acetyl-beta-D-glucosaminidase; Plt, platelet; RBC, red blood cell; T.chol, total cholesterol; TIBC, total iron-binding capacity; TP, total protein; UA, uric acid; WBC, white blood cell

**Fig. 2:**
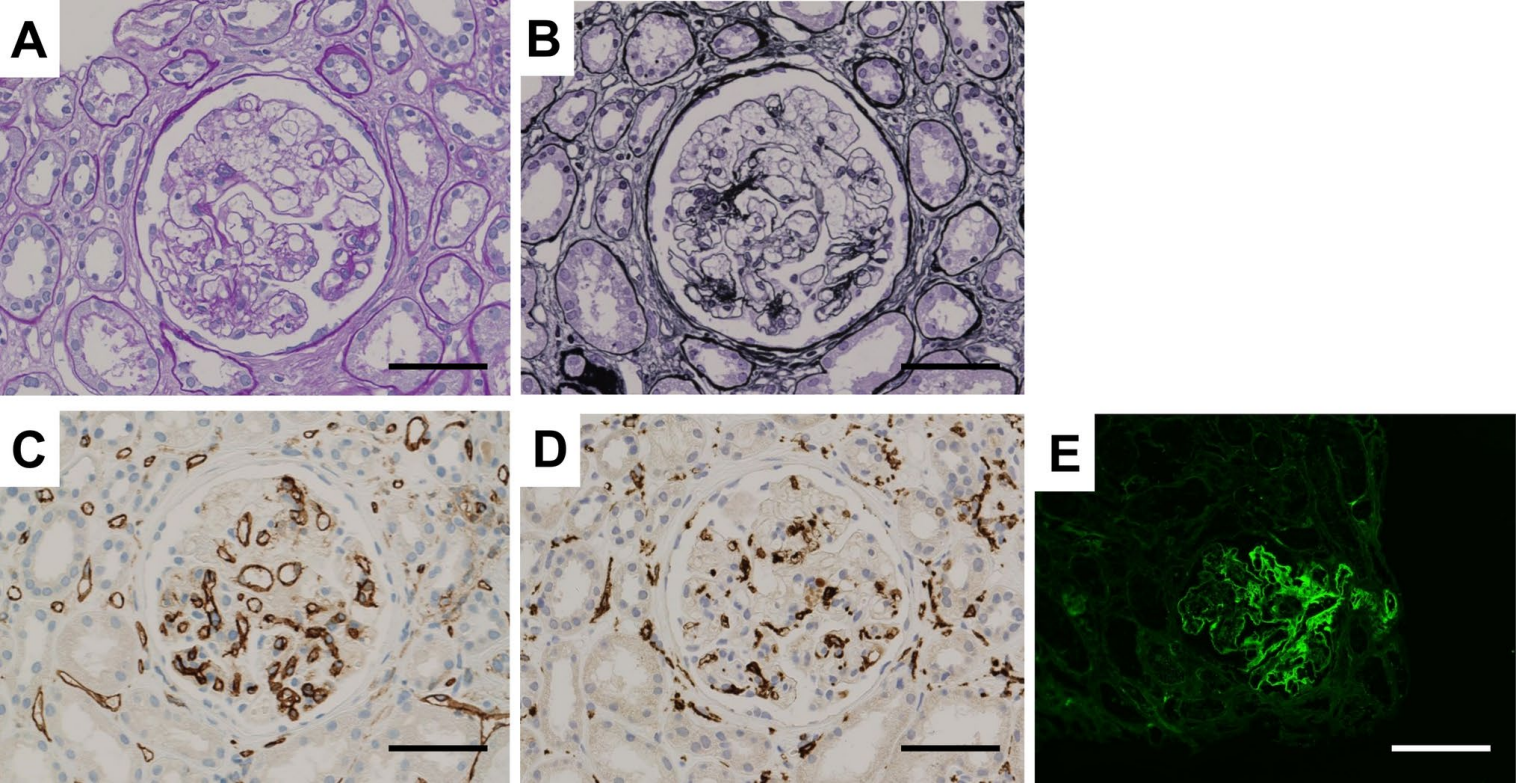
Light microscopic findings of renal biopsy. **A**: Periodic acid Schiff (PAS) stain, × 400. **B**: Periodic acid-methenamine silver (PAM) stain, × 400. **C**: Immunohistochemical staining for CD31. **D**: Immunohistochemical staining for CD163. **E**: Immunofluorescent staining for C4d. Scale bar, 100 µm

**Fig. 3:**
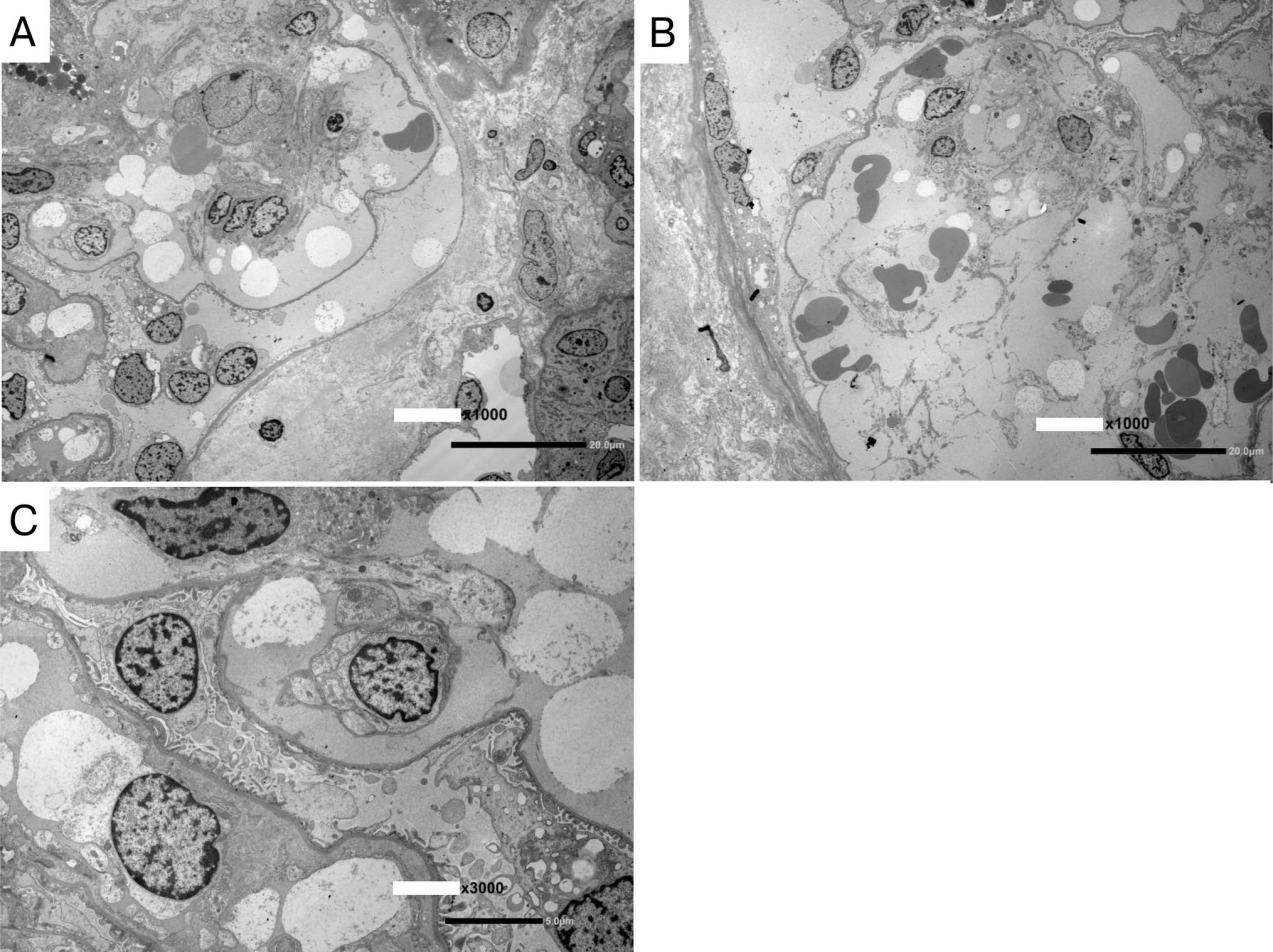
Electron microscopic kidney biopsy findings

A kidney biopsy was performed at 14 months after HSCT. The tissue sample contained approximately 30 glomeruli, including one with global sclerosis and nine with segmental sclerosis. Light microscopy revealed mild mesangial matrix expansion and mild mesangial cell proliferation. Most glomeruli exhibited segmental double contours of the basement membrane and marked mesangiolysis with massive capillary loop expansion, which was consistent with capillary microaneurysm formation (Fig. 2). No thrombi were identified within the glomerular capillaries. Tubular atrophy and interstitial fibrosis affected approximately 40% of the cortex and were accompanied by moderate mononuclear cell infiltration. Arteries and arterioles showed moderate intimal thickening and hyalinosis. Immunohistochemistry revealed negative staining for CD31 along the walls of capillary microaneurysms, suggesting desquamation of endothelial cells. CD163-positive foam cells were observed within the microaneurysms, suggesting macrophage infiltration. Immunofluorescence revealed no glomerular staining for immunoglobulins or complement components, whereas diffuse, segmental fibrinogen deposition was detected within the glomerular tufts and mesangial areas. C4d immunofluorescence showed diffuse positive staining in the glomerular capillaries as well as the arteriole. Electron microscopy revealed mesangiolysis and massive expansion of the subendothelial space associated with endothelial cell detachment (Fig. 3). On the basis of these findings, a diagnosis of HSCT-TMA, compatible with radiation nephropathy, was made.

Following the biopsy, the patient was treated with an angiotensin II receptor blocker (ARB). However, her proteinuria persisted at 2.0–3.0 g/gCr, and her serum creatine level continued to worsen.

## Discussion

TMA is clinically characterized by hemolytic anemia with fragmented red blood cells in the peripheral blood smear, thrombocytopenia, and elevated serum lactate dehydrogenase (LDH) levels [10–12]. HSCT-TMA is caused primarily by vascular endothelial injury resulting from a combination of chemotherapy, radiation therapy, nephrotoxic agents, infections, and GVHD [13–15]. Endothelial damage activates neutrophils, leading to the formation of neutrophil extracellular traps (NETs) and subsequent activation of the alternative complement pathway. Eventually, platelet activation promotes thrombus formation, contributing to the development of HSCT-TMA [16–18]. The kidneys are particularly vulnerable to endothelial injury, and patients with HSCT-TMA may present only with kidney manifestations, such as impaired kidney function, proteinuria, and hypertension [13]. Kidney pathology often reveals morphologic changes indicative of endothelial injury, including mesangiolysis, endothelial edema, subendothelial expansion, and glomerular capillary thrombi [13, 14, 19].

In the present case, kidney dysfunction and proteinuria emerged approximately six months after HSCT and gradually progressed. Although classic hematological features of TMA, such as hemolytic anemia with fragmented red blood cells, thrombocytopenia, and elevated LDH levels, were absent, HSCT-TMA was suspected on the basis of several risk factors, including TBI, CNI, TKI, and GVHD. Kidney biopsy revealed endothelial cell detachment in the glomeruli with associated subendothelial widening, along with mesangiolysis. In particular, the observed mesangiolysis was characterized by diffuse segmental capillary microaneurysms. Diffuse C4d positivity was observed in the glomerulus and the arteriole. These findings indicate that both endothelial and mesangial injury are very severe, which is consistent with the findings of HSCT-TMA. Renal arteriolar C4d deposition may reflect complement activation in HSCT-TMA [20], although this finding is not entirely specific and can be seen in TMA of other etiologies [21]. Although the presence of arteriolar hyalinosis raises the possibility of concomitant CNI vasculopathy, such remarkable mesangiolysis with diffuse capillary microaneurysms has not been typically reported in CNI–associated TMA, suggesting that CNI alone is unlikely to fully explain the glomerular lesion. In addition to experimental models, such mesangiolysis patterns have been reported almost exclusively in irradiated cases [22–28]. Therefore, we consider that the HSCT-TMA in this case was caused primarily by radiation nephropathy.

Mesangiolysis refers to degeneration of mesangial cells and degradation of the mesangial matrix. According to the morphological classification by Morita et al. [29], the mechanisms underlying mesangiolysis can be broadly categorized into direct injury to mesangial cells or secondary injury due to adjacent endothelial cell damage. The typical causes of direct mesangial cell injury include habu venom and anti-Thy-1 antibody nephritis, both of which have been described to cause significant ballooning. Therefore, mesangiolysis accompanied by microaneurysms, as observed in this case, is considered suggestive of mesangial injury. Secondary mesangial injury associated with endothelial damage is clinically prevalent, and CNIs are a salient cause. Mesangiolysis in this context often coexists with endothelial edema, subendothelial expansion, and thrombus formation in glomerular capillaries and arterioles. In this case, severe damage to the endothelium and mesangium was observed, suggesting that both contribute to the development of mesangiolysis.

Radiation causes cellular damage through direct DNA strand breaks and indirectly via reactive oxygen species (ROS) generation [30]. In animal models of radiation nephropathy, leukocyte adhesion to the glomerular capillary endothelium is followed by endothelial swelling and subendothelial plasma and leukocyte exudation [31], suggesting that radiation exposure causes endothelial cell injury. In this case, immunohistochemistry revealed the loss of CD31 expression in the walls of capillary microaneurysms, suggesting the desquamation of endothelial cells due to endothelial injury. Furthermore, experimental studies examining ROS-mediated damage have described the occurrence of mesangiolysis with microaneurysms, along with endothelial swelling and detachment from the basement membrane [32]. In studies using cultured rat mesangial cells have shown that ionizing radiation can directly induce mesangial cell apoptosis [33] and modulate profibrotic gene expression [34]. These findings suggest that both endothelial and mesangial cells are susceptible to radiation damage. Given the limited number of studies exploring radiation-induced kidney injury, this remains an important area for future investigations.

Radiation nephropathy typically presents with a latent onset, which manifests after a period of 6–12 months following radiation exposure. It can be managed with angiotensin-converting enzyme inhibitors or ARBs [35]. Sodium-glucose co-transporter-2 inhibitors may also be considered as a potential option because they have been shown to slow the decline of kidney function and reduce albuminuria in CKD patients with proteinuria [36, 37]. However, clinical evidence for treatment of radiation nephropathy is limited, and no specific therapies are available. Therefore, prevention is more important than treatment. Several studies have reported that limiting the radiation dose to less than 10 Gy by kidney shielding can reduce the incidence of kidney dysfunction after TBI [38, 39]. Additionally, fractionated TBI regimens with total radiation doses less than 12 Gy have been associated with a decreased risk [40, 41]. However, as in this case and several others [26–28], radiation nephropathy can still occur despite these protective measures. On the other hand, some patients do not develop radiation nephropathy even in the absence of kidney shielding. Genetic susceptibility may influence kidney sensitivity to radiation [42], although the exact mechanisms remain unclear. Further studies are needed to identify patient characteristics that predispose patients to radiation nephropathy.
